# Acknowledge the Elephant in the Room: The Role of Power Dynamics in Transforming Food Systems: Comment on "What Opportunities Exist for Making the Food Supply Nutrition Friendly? A Policy Space Analysis in Mexico"

**DOI:** 10.34172/ijhpm.2022.7382

**Published:** 2022-07-26

**Authors:** Angela Carriedo, Helen Walls, Kerry Ann Brown

**Affiliations:** ^1^Department of Health, University of Bath, Bath, UK.; ^2^World Public Health Nutrition Association, Peacehaven, UK.; ^3^Faculty of Public Health & Policy, London School of Hygiene & Tropical Medicine, London, UK.; ^4^College of Life & Environmental Sciences, University of Exeter, Exeter, UK.

**Keywords:** Mexico, Power, Food Systems, Food Policy, Sustainability, Inequity

## Abstract

A paradigm shift is required to transform food systems, so they are more equitable, environmentally friendly, and healthy. This requires acknowledging which factors change or maintain the status quo. In this commentary, we reflect upon the Cervantes et al study findings and discuss the role of power dynamics in transforming food systems. This is directly relevant to Mexico in terms of (*i*) relationships between food system actors; (*ii*) the role of socio-economic political context; and (*iii*) opportunities for policy coherence and transformative food systems approaches. We suggest that the power dynamics that drive the food produced, sold, and consumed should be recognised in all (inter)national governance decision-making. The 2021 United Nations Food System Summit – when interest groups were perceived to overly influence the summit proceedings – is an example of how neglecting the role of power dynamics can undermine and slow food system transformation.

## Introduction

 The coronavirus disease 2019 (COVID-19) pandemic together with a growing global concern regarding climate change has brought into sharp focus the fragility of food systems, and the vulnerability of populations to disrupted food supplies and food insecurity. The fragility of the food system is no surprise to the global health community who have long advocated for food system transformation to address sustainability and inequity in how food is produced, sold, and consumed.^1^

 Food systems represent the activities and outcomes of the food supply chain – the production, processing, distribution, retail, consumption and disposal or waste of food – and the contextual factors shaping these processes. Relationships between activities of the food supply chain and contextual factors (eg, economic or ecological factors) are complex and dynamic. Areas of the food system can also be in conflict, dependent upon whether food is being viewed as a tradable commodity, a human right, a health requirement, part of the local ecology, or a combination of the above.^2^ This makes designing and implementing any interventions to transform food systems challenging and at times unclear, unpredictable, or even contentious.

 Internationally agreed government targets, such as the United Nations 2030 Sustainable Development Goals (SDGs) encourage a coherent, whole-government approach to transformative change. These goals promote policy-making with simultaneous core values to improve health, inequity, and environmental sustainability. Yet, there is little guidance on how to achieve this policy coherence, and progress on SDGs has been slow, compounded by the COVID-19 pandemic, and the international community is not on course to universally meet food-relevant SDG, such as the eradication of hunger by 2030 in SDG 2 (eg, 2.37 billion people were without food or unable to eat a healthy balanced diet on a regular basis in 2020).^3^ This slow progress suggests that achieving policy coherence to improve food systems is challenging in practice.

 The Cervantes et al^4^ study explored pathways to transform the food systems’ status quo by examining how nutrition was framed in agricultural, economic, and health policy sectors in Mexico. They identified several issues that appeared to hinder food system transformation, including differences in priorities from actors representing different areas of/interests in, the food system. Future opportunities for policy coherence were also discussed through the establishment of the Intersectoral Group of Health, Food and Environment and Competitiveness (Grupo Intersectorial de Salud, Alimentación y Medio Ambiente, GISAMAC) in Mexico: a multi-sectoral initiative to develop food and agricultural policy. These factors are important considerations to change the status quo and transform food systems. The Cervantes et al study did not, however, address fully the elephant in the room – the role of power dynamics in transforming food systems.

## Changing Power Dynamics Between Mexican Food Actors – Will This Facilitate Food System Transformation?

 Despite power dynamics that could constrain policy-making, Mexican has been progressive with its food policy to target the country’s consumption of ultra-processed foods and nutrients of concern (eg, Mexico was the largest soft drink market in the world in 2014, with average consumption at 158 litres per capita per day, second only to Chile^5^). In 2014, a sugar sweetened beverage (SSB) tax was introduced, and in 2019, warning labels on packaged foods (for energy, sodium, refined sugar, and total fat) replaced industry-defined Guideline Daily Amounts on front-of-pack nutrition labelling (FOPNL) which aligned Mexico with mandatory FOPNL in the wider region (Chile, Perú, Argentina). The SSB tax and warning label polices were highly challenged by corporate actors.^6^ Nevertheless, the policies passed, largely due to the support of key policy-makers and advocates, for example, the Ministry of Finance supporting the policies in legislative chambers.^6^

 Corporate interests historically have had great influence in the Mexican food policy space. Policies over the last decade to alleviate food poverty, under the Ministry of Social Development (now Ministry of Welfare) were shaped and delivered by industry actors. For instance, in 2013, the government partnered with PepsiCo to implement a programme aiming to alleviate hunger, called Cruzada contra el Hambre. In addition, the Minister of Health was the former head of a Nestlé funded organization, and corporate actors were included in the committee established in 2015 to monitor obesity policy (including SSB taxation and FOPNL).^7^

 Cervantes et al^4^ noted, currently, nutrition is not embedded across all food and health relevant policies in Mexico. Their study identified policies in several government departments, including the Ministry of Social Welfare (Prospera, previously Progresa, and now cancelled) and the Ministry of Economy (United States, Mexico, Canada Agreement, USMCA, previously the North-American Free Trade Agreement, NAFTA), which did not prioritise nutrition and were more aligned with viewing food as a tradable commodity than a health requirement. In a neoliberal context, these government policies have been influenced by the participation of the private sector, including business associations and representatives of leading food and beverages corporations in the country. Inherent competing interests have, therefore, played a role in maintaining the status quo and/or ensuring limited damage to business interests.^8^

 In Mexico, as in other countries, corporate actors have strategically and systemically hindered and delayed public health efforts and have gained a legitimate and powerful position in both national and international policy venues.^9^ Fortunately, some actions to address power imbalances have been taken by civil society groups, academics, and activists over the last decade. For example, the “Via Campesina” was founded in Mexico in the early 1980s and new and more powerful alliances of claimholders have since emerged to support small-scale producers; help government to manage corporate engagement; highlight the risks of public-private partnerships; and support human rights in all policies.^10^ Reflected in all of these actions is a push, globally, for transparency and accountability in how policy is developed and implemented, and how food system transformation is being shaped or challenged by corporate actors, which have traditionally been powerful at influencing food policy in Mexico, and internationally.^7^

## How Can the Mexican Political and Economic Context Facilitate Food Systems Transformation?

 Understanding the socio-economic and historical context of food policy in a country or region is critical to achieving food system transformation. This context shapes traditional political and socio-economic infrastructure (eg, government department roles and political processes), policy priorities, values, and beliefs, as well as power dynamics in policy-making. Historically, food and agriculture policy-making in Mexico has largely been shaped by the post-second world war Green Revolution (~1940-1980), and inclusion as a pioneer country in the Rockefeller Foundation’s Mexican Agricultural Program. The primary aim of this programme was to ensure energy supply and meet hunger requirements by increasing food productivity and availability. This package of programmes opened the door to Mexico’s neoliberalist food policies by encouraging a rapid uptake of high-yielding maize and agrochemical inputs. These policies were more suited to large commercial farms that could afford the high upfront costs of seed and agrochemical inputs, rather than small-scale farmers.^11^ In 2018, the new presidential administration aimed to support small-scale farmers and protect indigenous staple crop species from imported patent seeds, monocrops, and the use of glyphosate. These policies have not yet been implemented, and it is unclear the degree of corporate pressure that has delayed any policy change.

 As seen with other low- and middle-income countries, Mexico’s participation in international trade agreements and global supply chains limits its ability to transform national food systems. NAFTA was signed in the early 1990s and coincided with rapid changes to the food environment and food consumption practices: moving from traditional Mexican diets to a steady increase of ultra-processed food consumption, including SSBs. Global trade agreements have been shown to delay actions in global health policy to alleviate non-communicable diseases.^12^ Power dynamics, therefore, play out at a global as well as a national stage.

 At the national level, Mexico is tackling a double burden of malnutrition. Traditionally Mexican health policies have targeted under-nutrition (eg, stunting, wasting, and micronutrient deficiencies). Progress has been made to improve wasting (low weight for height) with a prevalence of 1.5% in under 5-year-olds in 2020. Stunting (short height for age) and low birth weight, however, remain a problem (4.4.% prevalence of low weight and 15.5% prevalence of stunting in under 5-year-olds in 2020).^13^ This is alongside a growing prevalence and health burden related to over-nutrition. Mexico has disproportionately high rates of obesity: 78.0% of the adults are either overweight or obese; and 8.4% of under 5-year-olds as well as 38.2% of school children are overweight.^13^ Still policies rarely address malnutrition in all its forms ie, simultaneously tackling under- and over-nutrition. The mother and child health inter-government program, ESIAN, is an exception. ESIAN includes a component on obesity and hypertension, as well as covering micronutrient deficiencies and infant growth.^14^ Other than this programme, as Cervantes et al^4^ findings suggest, there is room for greater policy coherence between nutrition policies in the health sector, as well as greater coherence across all food and health relevant policies.

## Will Mexico Move Towards Policy Coherence and Transformative Food System Approaches?

 Currently, it suits many actors that areas of the food system are opaque. Moving towards policy coherence on nutrition and more widely transforming food systems to safeguard public and planetary health and avoid the exploitation of human, animal, or environmental resources, however, requires openly recognising the inter-connectedness and inter-dependency of different elements of our food supply chains. Taking this wider food systems perspective helps with identifying where there is policy coherence or conflict and acknowledges the power dynamics or different views of food (ecology, health, economy, human right) that drive policy decision-making and food system governance.^2^

 To date, no country has seemingly achieved a fully integrated food policy: establishing a shared political or national vision and policy coherence so that all policies complement and work towards the same end goal.^2^ Mexico now has the opportunity to consolidate a new government structure to facilitate policy coherence, avoid political silos, and adopt a food systems approach. The GISAMAC is a multi-sectoral initiative to develop and implement agriculture and health policies simultaneously, which is being co-ordinated by both ministries. This initiative also includes the collaboration of academics, activists, and intergovernmental organizations, and specifically includes policy framing on the human right to access food and water, whilst having strict policies to avoid participation of actors with conflict of interest, in line with recent Pan American Health Organization recommendations.^15^ Using a human rights view to inform food-related policy agendas has been successful in many countries, particularly when supporting children’s rights (eg, mandatory restrictions on food marketing practices in Chile, Mexico, Argentina and recently Spain).^16^

## Conclusion

 Cervantes et al^4^ achieve a detailed understanding of the political context for improving food systems in Mexico. Our main recommendation is to acknowledge the elephant in the room: the role of power dynamics in transforming food systems. To achieve any change, it is critical to disentangle and address the power dynamics driving the status quo of food systems and explore the influence of transnational food corporations and other policy actors at national, sub-national and global levels.

 Following the 2021 United Nations Food System Summit, many countries are in the process of developing ‘road maps’ on how national policies can help to achieve food system transformations. There have been criticisms that the summit encouraged countries to collaborate with powerful global food transnational corporations. The international community might consider to (*a*) follow the progress of the Mexican inter-secretariat and inter-sectoral collaboration in GISAMAC, which under its mandate has excluded corporate actors of any activity related to food and agriculture policy-making; and (*b*) monitor the degree GISMAC recommendations and actions align with the national Food System Summit Mexico ‘road map’ to transform food systems. This inter-secretariat collaboration can help provide insights on how power dynamics can best be managed given different and at times competing views of food will influence the degree future food systems can adapt to become more healthy, equitable, and environmentally sustainable.

## Ethical issues

 Not applicable.

## Competing interests

 Authors declare that they have no competing interests.

## Authors’ contributions

 AC, HW, and KAB were all involved in the conception and design, analysis and interpretation, drafting of the manuscript, and critical revision of the manuscript.
